# Qualitative study exploring the phenomenon of multiple electronic prescribing systems within single hospital organisations

**DOI:** 10.1186/s12913-018-3750-1

**Published:** 2018-12-14

**Authors:** Zamzam Ahmed, Yogini Jani, Bryony Dean Franklin

**Affiliations:** 1 Research Department of Practice and Policy, UCL School of Pharmacy, 29-39 Brunswick Square, London, WC1N 1AX UK; 2 The Centre for Medication Safety and Service Quality, Pharmacy Department, Imperial College Healthcare NHS Trust, Fulham Palace Road, London, W6 8RF UK; 3 Department of Clinical and Pharmaceutical Sciences, University of Hertfordshire, Hatfield, Hertfordshire AL10 9AB UK; 4 Centre for Medicines Optimisation Research and Education, Pharmacy Department, University College London Hospitals NHS Foundation Trust, 235 Euston Rd, London, NW1 2BU UK

**Keywords:** Electronic prescribing, Computerised provider order entry, Multiple electronic prescribing systems, Patient safety

## Abstract

**Background:**

A previous census of electronic prescribing (EP) systems in England showed that more than half of hospitals with EP reported more than one EP system within the same hospital. Our objectives were to describe the rationale for having multiple EP systems within a single hospital, and to explore perceptions of stakeholders about the advantages and disadvantages of multiple systems including any impact on patient safety.

**Methods:**

Hospitals were selected from previous census respondents. A decision matrix was developed to achieve a maximum variation sample, and snowball sampling used to recruit stakeholders of different professional backgrounds. We then used an a priori framework to guide and analyse semi-structured interviews.

**Results:**

Ten participants, comprising pharmacists and doctors and a nurse, were interviewed from four hospitals. The findings suggest that use of multiple EP systems was not strategically planned. Three co-existing models of EP systems adoption in hospitals were identified: organisation-led, clinician-led and clinical network-led, which may have contributed to multiple systems use. Although there were some perceived benefits of multiple EP systems, particularly in niche specialities, many disadvantages were described. These included issues related to access, staff training, workflow, work duplication, and system interfacing. Fragmentation of documentation of the patient’s journey was a major safety concern.

**Discussion:**

The complexity of EP systems’ adoption and deficiencies in IT strategic planning may have contributed to multiple EP systems use in the NHS. In the near to mid-term, multiple EP systems may remain in place in many English hospitals, which may create challenges to quality and patient safety.

**Electronic supplementary material:**

The online version of this article (10.1186/s12913-018-3750-1) contains supplementary material, which is available to authorized users.

## Background

Adoption of health information technology (HIT) in English secondary care organisations began in the 1980s. At that point, adoption was generally ‘bottom-up’ which meant that individual hospital organisations selected which HIT system(s) to implement. In early 2000, the English government started a ‘top-down’ strategy through the national programme for IT, aiming to deliver an integrated solution nationally [1]. The program was dismantled in 2011 due to delays in delivery and attributed costs. The English government subsequently offered financial incentives for NHS hospital organisations to adopt electronic prescribing (EP) systems and other technology [2]. In October 2014, a ‘five-year forward view’ to revolutionise the English NHS was published [3]. This acknowledged the drawbacks of the previous government information technology (IT) strategy and proposed a new approach with the aim of achieving interoperability between NHS systems and services. Against this background of shifting governmental IT strategy, a diverse picture of EP use has developed in NHS organisations [4].

A previous cross-sectional census of EP systems in 165 English NHS hospital trusts revealed that 69% of 101 respondents had some form of EP in place, with more than half of these reporting multiple systems, ranging from two to six [4]. The definition of EP in the survey was any form of EP operational in at least one ward or clinical area; this included EP modules that formed part of a wider HIT system.

Several systematic reviews have examined the impact of EP or computerized physician order entry use on medication errors and patient safety [5–8]. Multiple EP system use has been reported to cause medication errors due to miscommunication between systems [9]. However, the reasons for this phenomenon and its implications have not been fully explored. We therefore conducted a qualitative study to explore the perceptions of stakeholders about the reasons for multiple EP systems, the benefits and the potential challenges.

### Objectives

To describe the rationale for having multiple EP systems within a single hospital, and explore perceptions of stakeholders about the advantages and disadvantages of multiple systems, particularly including any impact on patient safety.

## Materials and methods

Study sites were selected from the previous census respondents [4]. The inclusion criteria were (1) hospitals that reported use of two or more EP systems and (2) consent previously given to be contacted for follow-up. We excluded outpatient EP systems as only two systems (2%) were reported in the census [4].

**Fig. 1:**
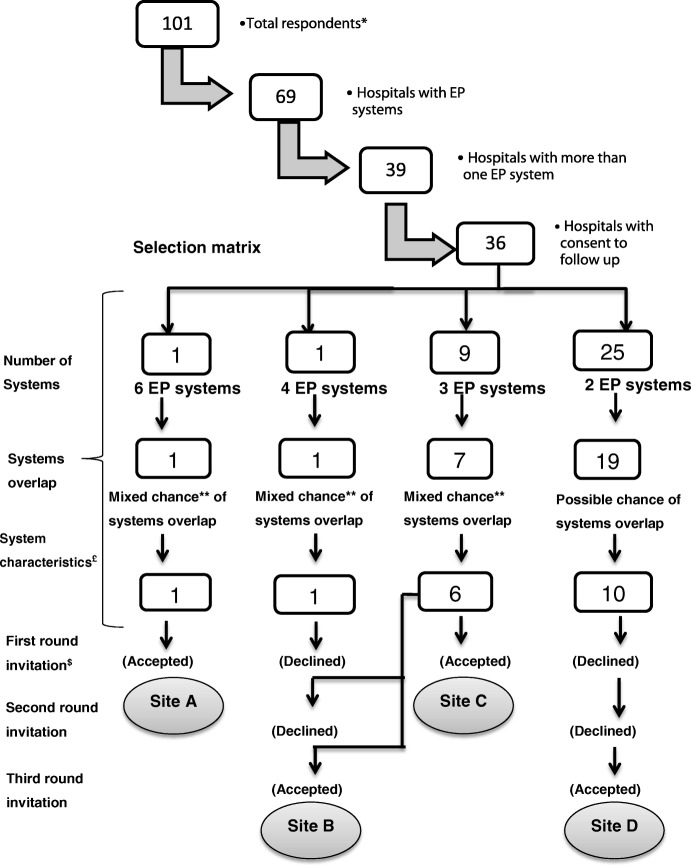
The screening and selection process of study sites. EP: electronic prescribing system.*The previous census respondents (Ahmed et al. 2013 The use and functionality of electronic prescribing systems in English acute NHS trusts: a cross-sectional survey. PLoS One, 8, e80378).** Mixed chance means that hospitals had system pairs with different likelihood of interaction. £ How systems were developed, types of prescribing, general vs. specialist systems, and prescribing for specific age groups. $ Hospitals were invited to participate, if invitation declined, another hospital was then selected and invited (a total of 3 invitation rounds were sent)

A decision matrix was created for the hospitals that met these inclusion criteria with the aim of achieving a maximum variation sample based on: number of EP systems in the hospital, likelihood of overlap (the extent to which multiple systems may be used for the same patients, and/or by the same individual healthcare professionals), and characteristics of the EP systems. System characteristics were how they were developed (commercial, in-house or hybrid), type of prescribing (inpatient and/or discharge), general versus specialist systems (chemotherapy, renal, critical care, etc.), and prescribing for specific age groups. Likelihood of systems overlap was based on a series of assumptions (Additional file 1). The process of site selection is summarised in Fig. 1.

The original respondents from the selected sites, predominantly pharmacists, were invited to participate. A snowball sampling technique was then used to recruit further participants from different professional backgrounds such as nurses, doctors and IT staff, particularly aiming to recruit potential users and/ or managers of more than one EP system.

**Fig. 2:**
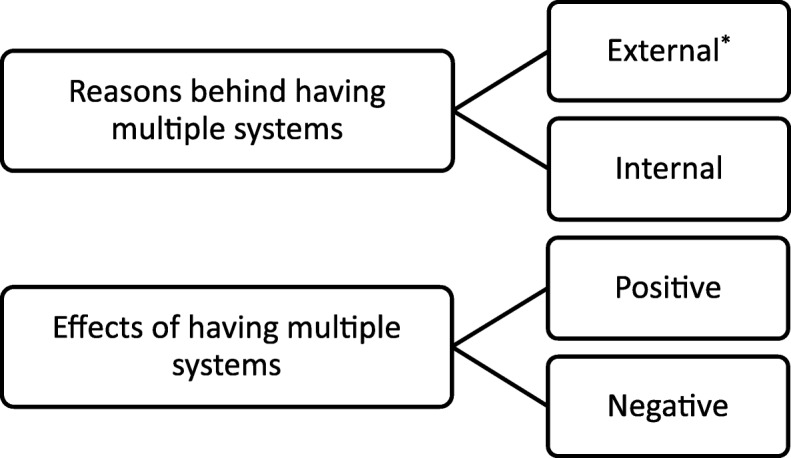
The initial conceptual framework developed to guide the interview and analysis process. * External to the organisation or hospital

An a priori framework (Fig. 2) was developed and refined following piloting to address the objectives and inform the interview guide (Additional file 2). A participant information sheet and consent form were emailed to participants prior to interview. Interviews were conducted via telephone or face to face between September 2014 and January 2015. Interviews lasted a maximum of 45 min. Interviews were audio recorded then transcribed verbatim.

Framework analysis, an approach that supports systematically reducing the data and analysing it by case and by theme, was used to organise and analyse the data [10]. Each hospital was considered to be one case. Transcribed interviews were uploaded to NVivo (version 10) and coded by researcher 1 to identify both predefined and emerging themes. Researchers 2 and 3 reviewed the coding of a sample of the transcripts. Researcher 1 reviewed all the codes and grouped them into themes. Emerging codes and themes were then refined iteratively. The resulting coding tree and each stage of refinement of the framework were reviewed by researchers 2 and 3. Data were charted in framework matrices using NVivo (version 10, QSR International); these were then used for analysis and interpretation. NHS ethics approval was not required under Health Research Authority regulations as the study involved the use of non-sensitive, anonymised interview procedures where participants were not defined as “vulnerable”. The study was approved by UCL research ethics committee.

## Results

The decision matrix revealed that four hospitals were required to meet maximum variation sample. Contacts in eight hospital organisations were approached sequentially, of whom four agreed to participate (Table 1). Site A had six EP systems (all commercial), site B had three EP systems (two commercial and one in-house), site C had two EP systems (one commercial and one in-house) and site D had three EP systems (all commercial).

**Table 1:** Overview of the study organisations, systems and the interviewees

	Number of acute hospitals	Number of EP systems	Types of systems	Integration between clinical & information technology services	Interviewees
Site A	1	6	All commercial	No	3 senior pharmacists
Site B	1	3	Two commercial, one in-house	Yes	2 senior pharmacists 2 senior doctors 1 senior nurse (super-user)
Site C	1	2	One commercial, one in-house	Yes	1 senior pharmacist
Site D	2	3	All commercial	Yes	1 senior pharmacist

*EP* Electronic prescribing

A total of 25 participants were invited, of whom ten agreed to take part (Table 1). Interviewees from sites A and D felt that due to the nature of the EP systems they used, only those clinical staff working across different specialities, such as pharmacists, were exposed to more than one system. Therefore, only pharmacists were interviewed. Of the six IT staff approached across four sites, none agreed to take part. No alternative sites were approached due to restrictions on time and research team capacity.

**Fig. 3:**
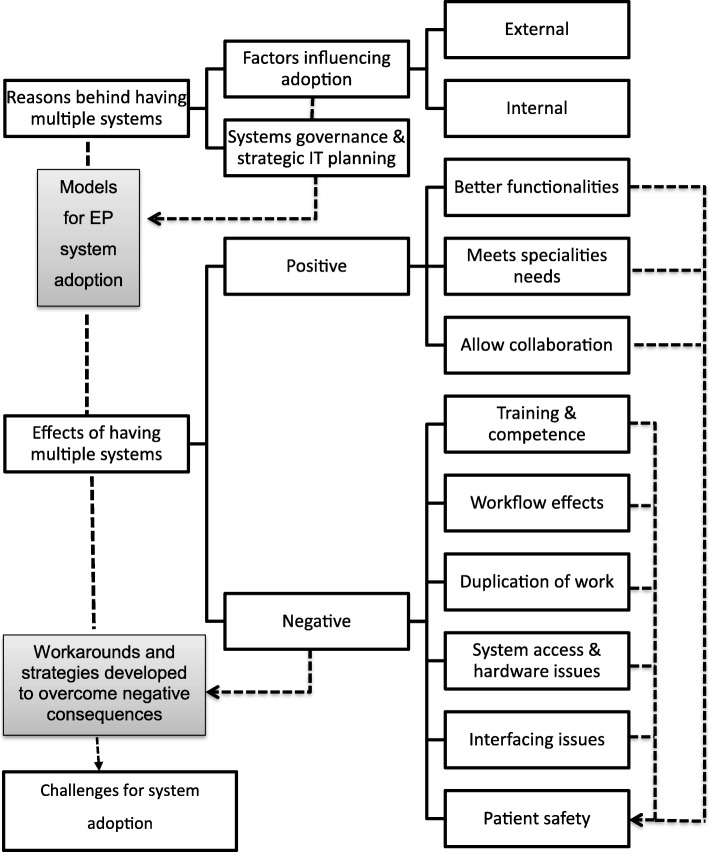
The expanded conceptual framework following analysis. EP: electronic prescribing. IT: information technology. Black boxes represent the extended conceptual framework of the study. Grey boxes represent new themes emerged from the study. Arrows indicate relationships

Figure 3 presents the final framework developed during the study. Results are next presented in the same sequence as in this expanded framework.

### Reasons for having multiple electronic prescribing systems

Two main themes emerged for having multiple EP systems: different drivers and models for system adoption, and level of systems governance and strategic IT planning.

#### Models of EP system adoption

Drivers of system adoption included funding mechanisms, system ownership and the stakeholders involved. Mapping these factors suggested that study hospitals had three models of adopting EP systems (Table 2). These models often ran concurrently within the same hospital, leading to multiple systems. The first was organisation-led adoption. Systems falling under this category were often used on a large scale within a hospital or organisation and were driven by local necessities and/or national drivers.

**Table 2:** Models of EP systems adoption

Organisation-led systems adoption	Clinician-led systems adoption	Strategic clinical network-led systems adoption
• Large scale use • Driven by local necessities and/or national drive • Governed by IT department • Example: A hospital wide discharge system or ePMA system	• Limited use (area or group of patients) • Driven by speciality needs • Supports a complete clinical pathway (not only prescribing) • Governed by the speciality • Example: ICU system	• Limited use (area or group of patients) but shared between hospitals • Driven by strategic clinical network • Supports a complete clinical pathway (not only prescribing) • Governed by speciality across hospitals • Example: Cancer System

*IT* Information technology, *ICU* Intensive care unit, *EP* Electronic prescribing, *ePMA* Electronic prescribing and medicines administration

The second adoption model was clinician-led. Such systems were introduced by a specific clinical speciality and limited to a clinical area and/or used for a specific group of patients, such as intensive care unit (ICU) systems. These systems were typically designed to support a complete clinical pathway for patients. Interviewees suggested that these systems were often introduced for benefits other than those expected from EP alone:

***‘…..*** *they [speciality systems] all bring benefits in addition to the prescribing abilities, so their systems are bespoke and built for that speciality. So for example the orthopaedic one will collect data for the bone registry and populate the letters, you can do bits in theatres and it will populate the letters for the stuff that’s relevant to that speciality rather than being a ‘does all’ but does a lot very well as that speciality would like it done, I guess. So, the systems have e-prescribing as a part of the package but that’s not the only thing that they do, so like an add-on I guess’*

Interview 2, pharmacist, site A

The third model of EP adoption was strategic clinical network-led EP adoption. All systems that fell under this category were cancer systems. These were similar in function to clinician-led systems, but shared between multiple hospitals. The choice of system was dictated by the specific cancer networks that hospitals were linked to.

#### System governance and strategic IT planning

IT department involvement ranged from full control to just providing technical support depending on the system and organisation. Some interviewees perceived there to be a clear overall IT strategy in their organisations; others reported lack of organisational IT planning. Some felt that IT departments did not take the lead in influencing the choice and introduction of EP systems, and were generally seen as ‘technical support’. A ‘best of breed’ approach, i.e. adopting multiple standalone systems or software applications designed to be used for individual specialties or areas [11], consequently changed over time giving way to organic growth of systems in one hospital:

*‘I think if you were to ask we would say that we were going for a best of breed approach where we pick the best piece of software but the reality is that it’s evolved as time has gone by so we’ve sat there and we’ve thought we need a renal information system and we’ve bought one and then we’ve thought we need a discharge letter and we’ve bought one. So it’s been the way that nobody’s ever really had a strategic plan about how we develop things I think, it’s just happened and then it’s been a question of trying to get things to talk to each other at the end’*

Interview 4, pharmacist, site A

Conversely, some hospitals had a strong, clearly defined IT strategy as well as integrated clinical and IT services. Interviewees reported project teams including clinical and IT staff working together, as well as strategies developed to overcome some of the negative aspects of having multiple systems, as described later.

### Effects of having multiple EP systems

Positive as well as negative effects were reported from the patient, user, and organisational perspectives.

#### Positive effects

One of the main reported advantages was providing better functionalities such as in-built safety features. Using bespoke speciality systems provided unique prescribing support in specific clinical areas as well as extra benefits such as fluid monitoring and balance chart support in ICU systems. Interviewees highlighted that the functionalities and benefits of bespoke systems may be challenging to replicate in a general prescribing system.

*‘…….having bespoke systems, so if we think about the chemotherapy systems it does do a lot more and it is very set up to manage chemotherapy protocols which would be very difficult to do within [system 1; electronic prescribing and medicines administration system], so it really has been built to deal with that kind of prescribing. It also has a lot of other functionality around scheduling and making appointments so that the day unit can keep their diaries. Then again, that would be quite hard for us to build into [system 1] in a way that works as well as it does for [system 2; ICU system] so bespoke systems will always do, will always work really well for that bespoke area and I think that is probably the key benefit’*

Interview 1, pharmacist, site B

The multiple EP system approach enabled end-users to meet their clinical speciality group needs, an advantage often perceived to be unattainable when adopting integrated systems as compromises often had to be made.

Furthermore, adopting multiple smaller systems allowed for an opportunity to align forces with other hospitals such as sharing cancer systems between hospitals. Such collaboration allowed spreading the load of systems management between linked hospitals as well as sharing expertise and knowledge. Interviewees felt collaboration was effective in niche clinical areas; sharing bigger (hospital-wide) systems with partners was likely to be problematic.

#### Negative effects

Many perceived disadvantages of having multiple systems emerged. If individual systems were used within discrete clinical areas, these disadvantages were generally limited to individuals or staff groups working across different disciplines such as pharmacists who needed to interact with several specialist systems in their day to day practice. Conversely, negative effects of having multiple EP systems seemed more prominent if at least one of the systems was hospital-wide.

##### Training and competence

Many reported difficulties related to training staff on multiple systems, especially at induction and if face-to-face training was involved. This was due to the large numbers of staff trained on various systems. Although e-learning was sometimes used, there was some resistance to it from staff who preferred to learn ‘on the job’. Customised training packages had to be developed depending on user role and access limits within the system.

Interviewees reported that staff had to learn a lot about each system in a relatively short time making it difficult for staff to retain much from training.

*‘It’s two things you have to learn, I think the more information you have to learn the more chance there is of mistakes and given that we have a high turnover of junior staff I think it’s a lot easier if they just have to learn one thing once’*

Interview 7, senior doctor, site B

There were challenges related to training on systems managed by other hospitals such as cancer systems, as often staff had to travel to other organisations for training.

Since some staff used systems sporadically, they could lose access because passwords expired or were forgotten, but more critically they felt less competent using the systems. Such issues were particularly problematic during out-of-hours and weekend coverage. Senior clinicians were less exposed to some systems and likely to delegate medication orders to junior staff.

‘*We have become dictators. We give the order then it’s not our problem anymore, it’s someone else’s problem. Junior doctors will have to sort out the orders while earlier I could have done some prescribing myself’*

Interview 8, senior doctor, site B

Locum staff were also affected by access and competence issues. Interviewees mentioned practices such as sharing passwords with locum staff and shifting IT related tasks to substantive staff if locums were unable to handle multiple systems.

*‘It’s apparent now if we have locums that really if they don’t know the hospital and the systems they are essentially fairly useless because somebody else has to look after all the IT input. IT is actually quite an important part of our working lives and the simpler and more error free it is, the better it is and I think two systems doesn’t really promote that.’*

Interview 7, senior doctor, site B

##### Effect on workflow

People changed some of their workflow to accommodate multiple system use. It seemed that workflow changes were more problematic at the start but then staff adapted to the new ways of working.

*‘People have had to change their way of working. So you might do something in a particular order but actually now that we’ve got [multiple] systems in place you might have to do it in a different order or you might have to approach your tasks in a slightly different way. So, where possible we have tried to outline ways to do that but what you find is that users actually find their own way to do it.*

Interview 1, pharmacist, site B

Although rare, workflow issues were more serious when data for the same patient were spread between two electronic systems. Staff had to log in to two different systems and locate the same patient’s records to prescribe, which was not only cumbersome but also risky as this may introduce errors.

*‘You would prescribe your anaesthesia in [anaesthesia system] and when you want to give a bolus of a drug post-op [post-operative] you have to go and login into ePMA [electronic prescribing and medicines administration]’*

Interview 7, senior doctor, site B

##### Duplication of work

Multiple systems could result in increased work volume due to duplication. This was at a patient level, such as entry of patient data in different systems, as well as system level tasks such as maintaining drug catalogues.

*‘The other obvious disadvantage with the bigger systems is that you’re having to maintain multiple catalogues and that’s going to be an issue between the ITU [intensive therapy unit] system and the main electronic prescribing system when we have it, that you’re going to be having to update and maintain the catalogue twice with your formulary decisions twice’*

Interview 9, pharmacist, site D

##### Systems access and hardware issues

Password burden was one of the prominent issues raised, with interviewees reporting difficulties in remembering multiple EP system passwords, as well as passwords for other systems. Using similar or sequencing passwords as well as noting them in smart phones or diaries were strategies used to overcome such challenges:

‘*The passwords I have at the hospital, I have my NHS password, my hospital password, my [system 1] password, my [system 2] password, we were counting, might get a university password, I have about 7 passwords in the hospital. I make notes on my [smartphone] of my current passwords and I now I tend to cross-populate, I used to have separate ones for all of them and now I tend to … the first one I change, I just change them all to the same password and then when it’s triggered again do the same thing, which I’m sure is not what you’re meant to do’*

Interview 7, senior doctor, site B

Procedures to allocate and renew passwords had to be created in order to guarantee all staff were able to access systems when required. Hardware requirements also had to be assessed carefully to meet demands of accessing multiple EP systems:

*‘…then making sure that the right people have got the passwords at the right time to be able to get into the system. You’ve got to make sure that you have got enough equipment available for everybody and that all the programs work on the same equipment, so that you are able to do everything from the one terminal if you need to.*

Interview 1, pharmacist, site B

##### System interface issues

All interviewees reported that attempting to interface systems was difficult. A possible explanation given by some of the interviewees was the complexities of some systems or the differences in the coding within each system. Therefore, what in principle seemed a straight forward process was actually far more complicated.

##### Patient safety

One of the main concerns about multiple systems was the effect on patient safety. Many of the reported disadvantages could affect patient safety:

*‘it is making sure people know that there is information in different places, making sure that they are trained, making sure nothing gets missed, making sure that prescribers are putting the drugs into the systems being used in that area, which I think can be difficult and then obviously if you have got a new system there are training issues and making sure that people are able to use the system effectively to deliver patient care, so I think there are definitely risks. It would be much less risky if you just had one system but we have to just find ways to mitigate those risks’*

Interview 1, pharmacist, site B

Having patient data spread across multiple EP systems hindered healthcare professionals from obtaining a complete picture of the patient journey. For instance, a doctor or nurse treating an outlier patient (a patient in another specialty’s ward due to lack of beds) might not be aware of important patient-related clinical data if they have no access credentials to a specific EP system. Some interviewees reported incidents where diagnosis of a newly admitted patient was delayed because of a ‘black hole’ in the patient prescription records:

*‘I think we’ve had a couple of occasions where a patient has been admitted, they’re generally unwell and it’s taken a little while for everybody to piece together the puzzle to say actually this patient’s getting this type of care and therefore there is a prescription and this is what they’re being prescribed and it’s happening somewhere else in our organisation but we can’t readily see that record’*

Interview 9, pharmacist, site D

Duplication of patient data in various systems was identified as another potential clinical risk. Slightly different information may be documented in each system:

*‘I think also there is another issue actually around duplication of information, so do people need to record things across different systems or can they put it in one place and expect that it will be found, and actually we don’t want people to have to duplicate stuff because we might get a slightly different story in each system. You want it recorded once and then for people to know where to find It’*

Interview 1, pharmacist, site B

In some instances, systems were not completely paperless as supplementary paper-based records were also required. Therefore, healthcare professionals were faced by a mixture of paper charts and data spread across multiple EP systems.

In-built safety features of systems sometimes introduced risks, especially when healthcare professionals were accustomed to a certain feature that was available in some systems but not in others.

*‘You may get used to a system doing a certain thing when you move to the other system and it doesn’t do it, that could create a risk because in your other system it’s automatically checking.’*

Interview 9, pharmacist, site D

### Overcoming negative consequences of multiple systems

Staff developed various strategies and workarounds to reduce the disadvantages of multiple systems use, improve user experience, and improve patient safety. Strategies and formal workarounds were usually reported by hospitals that had integrated clinical and IT services. For example, in site C, both EP systems were linked to the patient administration system. Staff created a one-way allergy data feed from their main EP system to their chemotherapy system. However, they reported that setting up this interface had been complex.

‘Dummy prescriptions’, i.e. flags alerting healthcare professionals to other prescriptions existing on paper and/or other electronic systems, were another example of a workaround. All hospitals were also exploring the introduction of a ‘single sign on’ to alleviate password burden of their staff and improve user experience.

#### Challenges for system adoption

Managing users’ expectations about systems linkage was another emergent theme. Achieving comprehensive linkage between EP systems was perceived to be challenging as interviewees acknowledged the difficulties of interfacing multiple systems. Interviewees highlighted staff frustrations due to lack of integration between systems, particularly as their expectations were influenced by their standard of IT use in day-to-day life.

*‘I think that it’s the user’s expectation that they expect the systems to talk to each other and they don’t and I think that’s hard to manage, people saying “well, why doesn’t the blood result feed into this one?” and you say that there is no link, you actually do have to look in this other place for it, so there is definitely some difficulty around managing expectation*’

Interview 1, pharmacist, site B

*Oh yes. I think it’s really difficult with IT in the NHS because of what we know we can have just in our general day to day life and how we see systems working in everything that we do and we’re so used to IT.… when you then try to apply that standard, that expectation to what we can achieve in NHS systems it’s really frustrating that it’s so difficult to do the same thing’*

Interview 9, pharmacist, site D

Interviewees raised some issues around EP systems’ capabilities. It was suggested that advances in HIT were not keeping up with the rapid changes of healthcare. Therefore, some systems were not able to support management of patients with complex clinical requirements.

*‘… system at the moment struggles to deal with patients who have got several booked admissions for different types of care and that may be because when the system was first developed patients perhaps were only expecting them to be lining up to come and have one type of treatment. Now patients have so much co-morbidity and are living so long that we can expect them to have lots of things happening all at the same time and our electronic prescribing system doesn’t cope very well with that’*

Interview 9, pharmacist, site D

Interviewees reported lack of sufficient expertise to manage EP systems within the NHS. While IT departments provided technical support for EP systems, clinical input was provided by end-users. The separation between technical and clinical skills may have hindered appropriate system management. Interviewees highlighted the need for people with both clinical and IT knowledge.

*‘At the moment, the responsibility of the [hospital wide discharge system] kind of sits with IT. That can be problematic in terms of its good because it’s an IT system and therefore the technical aspects of what need to be done are within their remit anyway, but when you’re looking at it in terms of a clinical system that does cause a problem. We have a clinician who is nominated within the organisation as being the person who will take decisions around the [discharge] system, but again he’ll be doing it from a very clinical perspective rather than an IT […..] I feel that we will probably see a shift and maybe start to have some clinical IT posts more than pure IT posts that have got a responsibility in both areas’*

Interview 9, pharmacist, site D

## Discussion

To our knowledge, this is the first qualitative study to explore the perceptions of stakeholders about the reasons, benefits and challenges around use of multiple EP systems in the context of UK secondary care. The present study revealed that adopting multiple EP systems in NHS hospitals was generally not strategically planned. EP systems’ adoption was affected by various internal and external factors. Mapping these factors revealed three co-existing models of EP system adoption and considerable variation in system governance and IT department involvement. Having multiple EP systems was perceived to have some advantages, particularly in the context of systems used in niche clinical specialities. These bespoke systems supported not only prescribing but also other clinical processes, and enabled clinical speciality groups to meet their specific needs. A previous US survey highlighted similar findings in relation to the variations in the needs and electronic health record usage between general and specialist physicians [12]. Nevertheless, many disadvantages relating to multiple EP systems use were reported, many of which were perceived to affect patient safety. The main negative aspect we identified was fragmentation of the documentation of the patient’s journey. Our findings are consistent with literature citing safety risks associated with the lack of integration and interfacing of hospital health information technologies [13, 14]. On some occasions, healthcare professionals reported missing key information and/or not being able to obtain a full view of their patient’s record. Moreover, the same healthcare professionals sometimes dealt with electronic systems with different features, and/or paper systems, which led to reliance on or assumption of a certain level of decision support that was not borne out in practice.

The findings of the present study highlight the importance of integration between clinical and IT services at both the management level and in day to day clinical practice. The study suggests that hospitals with integrated IT and clinical services developed solutions to reduce some of the negative impacts of multiple EP systems. Recruiting staff with both clinical and IT expertise is advisable as it may help bridge the gap between IT and clinical services. The use of speciality bespoke systems in niche areas, such as cancer systems, was perceived to be irreplaceable. Therefore, efforts should be directed towards interfacing such systems or the development of suitable interoperable solutions to support patient safety in the face of multiple systems. Compromising workarounds such as sharing accounts or not logging off screens have been previously reported [15]. Our study revealed other practices used by end-users to reduce password burden, such as sequencing passwords and/or noting them on smart phones or diaries, which may compromise security. Therefore, the use of ‘single sign on’ system in hospitals with multiple IT systems should be encouraged. The findings of the present work established various drawbacks to multiple EP systems that may influence patient safety. Further work is needed to quantify and assess the clinical impact of multiple EP systems on patient safety. To eliminate unintended consequences of health IT and ensure patient safety it is vital to combine measurement of health IT safety with existing clinical risk management and safety program(s) in hospitals [16]. The use of models or frameworks [17] to guide implementation and evaluation of technology while maintaining coordinated care may also help address some of the issues raised.

The tension between hospital-wide and speciality systems has been previously described [18]. Previous literature has also cited the tensions and contradictions that arise due to implementing IT in healthcare [19]. Diversity of devices and approaches to implementing electronic patient record systems was also reported in a third of English survey respondents utilising a best of breed approach [20]. When interviewed, respondents cited some benefits of the best-of-breed approach, but also safety concerns similar to ours [20].

Our study is the first to specifically explore multiple EP systems use within single organisations, a phenomenon rarely described in the literature [9], in spite of the specific safety issues associated with EP. Our work exposed the three different models of EP systems adoption. Understanding these models is important for future IT strategic planning and system evaluation. While multiple EP systems use is widespread in England, the extent to which this is an issue elsewhere in unknown.

The study has several limitations. First, although our interviews provided rich data, we were only able to recruit a small number of participants across all sites. Second, our sites were purposively sampled to provide maximum variation. Although care was taken in sample selection, the final sample may not adequately reflect the study population. Third, despite efforts made to include healthcare professionals of various backgrounds, most of the interviewees were pharmacists, and we were only able to recruit one participant in two of the sites. Moreover, were unable to explore issues related to IT strategy as no IT representatives agreed to take part. Therefore, it is possible that the views and opinions voiced by interviewees may not adequately reflect those of other stakeholders. Fourth, the present study was not designed to explore the full range of workarounds [21–25] resulting from multiple EP systems and it is likely that others exist. Fifth, although every effort was made by the reviewer to probe for both positive and negative aspects of multiple EP systems use, there were more negative implications cited by the interviewees. This may reflect their experience, but could also reflect a tendency to focus on the negative aspects in relation to patient safety. Finally, it was sometimes difficult to ascertain if issues reported by interviewees were related to EP use in general or were specific to multiple EP systems. However, every effort was made by the interviewer to probe as to whether effects were attributable to multiple EP systems.

## Conclusion

The complexity of EP systems adoption may have contributed to the phenomenon of multiple EP systems in NHS hospitals. Three co-existing models of EP systems adoption in hospitals with multiple EP systems were identified. As well as the perceived benefits of multiple EP systems particularly in niche clinical specialities, many disadvantages were described. Hospitals with integrated clinical and IT services described various strategies used to mitigate negative aspects of multiple systems use. In the near to mid-term future, multiple EP systems use is likely to remain in place, which creates challenges for the NHS workforce and for patient safety.

## Additional files

Additional file 1: List of assumptions used to determine potential likelihood of interactions/ overlap between systems. A series of assumptions utilised to determine the Likelihood of systems overlap which was used in the decision matrix created aiming to achieve a maximum variation sample. (DOCX 14 kb)

Additional file 2: Interview guide- Multiple EP systems study. A list of the questions and prompts used in the interviews conducted in the study. (DOCX 17 kb)

## Abbreviations

EP: Electronic prescribing
HIT: Health information technology
ICU: Intensive care unit
IT: Information technology
NHS: National Health Service
UCL: University College London
