# Supplementary material for: Qualitative study exploring the phenomenon of multiple electronic prescribing systems within single hospital organisations
Source: BMC Health Serv Res. 2018 Dec 14;18:969. doi: 10.1186/s12913-018-3750-1 (PMC6295095; doi:10.1186/s12913-018-3750-1)
Supplement: Supplementary file 1 — List of assumptions used to determine potential likelihood of interactions/ overlap between systems. A series of assumptions utilised to determine the Likelihood of systems overlap which was used in the decision matrix created aiming to achieve a maximum variation sample. (DOCX 14 kb) [file 12913_2018_3750_MOESM1_ESM.docx]

List of assumptions used to determine potential likelihood of interactions/overlap between systems

**Scenarios with high likelihood of interaction**

- Hospital wide inpatient system and an ICU system
- Hospital wide inpatient system and a separate discharge system
- Hospital wide inpatient/discharge system and an ICU system
- Chemotherapy system and a second, separate, chemotherapy system
- Discharge system and a second, separate, discharge system*
- Hospital wide inpatient/discharge system and a separate discharge system*

**Scenarios with possible likelihood of interaction depending how the systems are used locally**

- Hospital wide inpatient system and a chemotherapy system
- Hospital wide inpatient/discharge system and a chemotherapy system
- Hospital wide discharge system and a chemotherapy system
- Chemotherapy system and an ICU system
- Chemotherapy system and a renal system
- Renal system and an ICU system
- Renal system and an outpatient system
- Renal system and a discharge system

**Scenarios of low likelihood of interaction**

- Hospital wide discharge system and an ICU system
- Hospital wide discharge system and accident and emergency system

* (The likelihood interaction of two discharge systems was considered high unless the two systems were used for prescribing for two different patient populations. For example, interaction likelihood of two discharge systems used for adults & paediatrics respectively was considered to be low. Interaction likelihood of two discharge systems used for different clinical areas e.g. one for mental health and one for hospital was considered as possible).

ICU: intensive care unit.
