# Supplementary material for: Qualitative study exploring the phenomenon of multiple electronic prescribing systems within single hospital organisations
Source: BMC Health Serv Res. 2018 Dec 14;18:969. doi: 10.1186/s12913-018-3750-1 (PMC6295095; doi:10.1186/s12913-018-3750-1)
Supplement: Supplementary file 2 — Interview guide- Multiple EP systems study. A list of the questions and prompts used in the interviews conducted in the study. (DOCX 17 kb) [file 12913_2018_3750_MOESM2_ESM.docx]

## Interview guide- Multiple EP systems study

|  | **Questions** | **Prompts and probes** |
| --- | --- | --- |
|  | The interviewer will briefly explain the aim of this research then ask the interviewee if they’ve have read the information leaflet and answer any questions they may have. Verbal consent will be then requested. |  |
| **Background** | **Q1.** Can you tell me a bit about your professional background?  **Q2.**  It was reported in a survey done in 2011 that you had more than on electronic prescribing (EP) systems in the hospital; How many EP systems are currently operational within your hospital?  **Q3.** What are these systems and in which clinical areas are they used?  **Q4.** Are any of these systems linked to each other? | - *Current role in the Trust* - *Years of experience* - *Previous work in other trusts* - *Any past experience with EP either as a user or in development* - *Which system, what aspect they use* |
| **Reasons behind having multiple systems** | **Q5.** What were the reasons/factors leading to have more than one system? | - *Prompt about legacy systems and/or specialist systems.* - *Prompt about the ‘organic growth’ of the organisation.* - *Prompt about IT role, link with clinical staff, clinical staff involvement in IT projects etc.* - *Government policy, NHS funds example: discharge systems to meet CQUINs, funding for cancer care etc.* - *Championship of staff in certain clinical areas* |
| **Safety aspects** | **Q6.** Do you use any EP systems in your work? If yes, could you tell me more about that?  Q7. Can you think of any health care professionals using more than on system or patient scenarios requiring the use of one more system?  **Q8.** In your opinion, what are the strengths and weaknesses of having more than one EP system?  **Q9.** Have you had any concerns about prescribing/administering/dispensing *(depending on the role of the staff member)* through the system/s, its' safety or effectiveness?  **Q10.** Can you describe the main difficulties or challenges you've experienced so far, if any? | - *patient safety, staff training issues, workflow effects* - *Prompt about clinical areas: wards were 2 systems are in use* - *Prompt about staff eg locums, pharmacy weekend coverage, nursing covering other wards* - *Groups of patients or clinical scenarios: cancer patient transferred to ICU* - *Prompt about centralised care e.g. discharge prescriptions of stroke patients etc.* |
| **Identifying potential future interviewees, and wrap up** | **Q13.** Could you think of any staff members or a clinical area that might use all or some of these systems on a regular basis? If so, then which staff or which area?  **Q14.** Could you suggest or nominate any of them to be invited for a similar interview?  **Q15.** Are there any other hospitals within your Trust having more than one EP system? If so, could you suggest any person to be invited for a similar interview?  **Q16.** Is there anything else would you like to tell me? |  |

EP: electronic prescribing, IT: information technology, NHS: National Health Service, CQUINs: the Commissioning for Quality and Innovation payments framework, ICU: intensive care unit.

Two questions relating to future procurement plans of EP system (question 12, and 13 as well as all related probing questions) were deleted from the guide as this section was not presented in this paper.
